# Resting state electrical brain activity and connectivity in fibromyalgia

**DOI:** 10.1371/journal.pone.0178516

**Published:** 2017-06-26

**Authors:** Sven Vanneste, Jan Ost, Tony Van Havenbergh, Dirk De Ridder

**Affiliations:** 1 School of Behavioral and Brain Sciences, The University of Texas at Dallas, Richardson, United States of America; 2 BRAI^2^N, Sint Augustinus Hospital Antwerp, Antwerp, Belgium; 3 Department of Surgical Sciences, Section of Neurosurgery, Dunedin School of Medicine, University of Otago, Dunedin, New Zealand; Banner Alzheimer's Institute, UNITED STATES

## Abstract

The exact mechanism underlying fibromyalgia is unknown, but increased facilitatory modulation and/or dysfunctional descending inhibitory pathway activity are posited as possible mechanisms contributing to sensitization of the central nervous system. The primary goal of this study is to identify a fibromyalgia neural circuit that can account for these abnormalities in central pain. The second goal is to gain a better understanding of the functional connectivity between the default and the executive attention network (salience network plus dorsal lateral prefrontal cortex) in fibromyalgia. We examine neural activity associated with fibromyalgia (N = 44) and compare these with healthy controls (N = 44) using resting state source localized EEG. Our data support an important role of the pregenual anterior cingulate cortex but also suggest that the degree of activation and the degree of integration between different brain areas is important. The inhibition of the connectivity between the dorsal lateral prefrontal cortex and the posterior cingulate cortex on the pain inhibitory pathway seems to be limited by decreased functional connectivity with the pregenual anterior cingulate cortex. Our data highlight the functional dynamics of brain regions integrated in brain networks in fibromyalgia patients.

## Introduction

Physiological pain typically originates from noxious stimuli that are able to trigger a neural response in pain-dedicated systems. In individuals with fibromyalgia, pain is experienced with noxious stimulation, but linked to abnormalities in central pain processing. Fibromyalgia is a disorder characterized by chronic (>3 months) complaints of spontaneous widespread pain in in all 4 quadrants of the body associated with fatigue not relieved by rest, poor sleep, distress, depression, or cognitive dysfunction [1].

The exact mechanism underlying this pain syndrome is not known, but increased facilitatory modulation and/or dysfunctional descending inhibitory pathway activity are possible mechanisms contributing to sensitization of the central nervous system [2]. Central sensitization is defined as an increased responsiveness of the central nervous system to a variety of stimuli such as pressure and temperature. This causes hyperalgesia, allodynia, and referred pain across multiple spinal segments, leading to chronic widespread pain [3]. Functional and structural neuroimaging studies have provided evidence for this hypothesis, demonstrating both structural and functional activity and connectivity changes resulting in enhanced pain facilitation in combination with defective inhibition of nociceptive signals, which augment pain perception [4, 5]. Activity changes have been identified in the insula, the anterior cingulate cortex, and the prefrontal cortex [6]. These areas are part of what used to be called the pain matrix, but has recently been shown to be non-specific for pain, as these areas are also activated by non-painful stimuli, auditory stimuli, and visual stimuli [7]. Based on previous studies looking into salience processing [8], it has been suggested that the pain matrix is actually a network involved in multimodal salience processing [9, 10]. Brain resting state functional connectivity changes were identified in the self-referential default mode network and the executive control network in fibromyalgia patients [6, 11–15]. These network changes in fibromyalgia are similar to what was found in chronic back pain patients, which was interpreted as a lasting effect of pain on brain function [16, 17]. A deficiency of the pain inhibitory pathways, which critically involve the pregenual anterior cingulate cortex (pgACC) has been found in fibromyalgia patients as well [4, 18]. In summary, functional imaging data suggest fibromyalgia is a clinical syndrome associated with a brain dysfunction related to an increase in salience attached to pain and a deficiency in pain inhibitory mechanisms.

In the current study, we examined neural activity associated with fibromyalgia using resting state source localized EEG. The primary goal of this study was to identify a fibromyalgia-related neural circuit that could account for these abnormalities in central pain. The second goal was to gain a better understanding of the functional connectivity between default and the executive attention network in fibromyalgia. In addition, we were able to explore potential abnormalities in different frequency bands using EEG. These bands are associated with different neural mechanisms, and we compare these findings with previously published results from other neuroimaging modalities.

## Materials and methods

### Subjects

A total of 88 subjects participated in the study, including 44 patients with fibromyalgia (*M* = 46.33; *Sd* = 9.56) and 44 healthy control subjects who were matched for age (*M* = 46.33; *Sd* = 9.56) and gender (males: 4; females: 40). The healthy control subjects were selected out of an EEG database (N = 264) that were collected by our group using the same equipment and room as the fibromyalgia group. Patients were consecutively recruited from outpatient clinics to obtain a homogeneous sample. All patients met the 2010 American College of Rheumatology criteria for fibromyalgia [19]. All patients were screened by a pain physician to positively diagnose them with fibromyalgia to become enrolled in this study. Patients presenting with pathologies mimicking the symptoms of fibromyalgia, as well as patients suffering from severe organic or psychiatric co-morbidity (except minor depressive disorder) were excluded from participation. None of the patients were suffering from cervicotrigeminal radicular pain or types of hemicrania. None of the patients enrolled were taking any medication or have a history of drug abuse. This study was approved by the local ethical committee (St-Augustinus Hospital, Antwerp, Belgium) and was in accordance with the declaration of Helsinki. Patients gave a written informed consent before the procedure. We have made the data available on figShare, with the accompanying DOI: https://figshare.com/s/a39acc64713d03706333.

### Questionnaires

#### 1. Pain questionnaires

*Fibromyalgia Impact Questionnaire (FIQ)*. The fibromyalgia impact questionnaire creates an inventory of the overall impact of fibromyalgia related symptoms on daily life. It is proven to be a well-designed questionnaire to measure the impact of fibromyalgia on the overall quality of life of patients. The maximum score is 100 and a higher score indicates a greater negative impact of the syndrome on the patient [20].

*Pain Vigilance and Awareness Questionnaire (PVAQ)*. The PVAQ measures the preoccupation with or attention to pain and pain changes and is associated with pain-related fear and perceived pain severity [21]. It consists of 16 items (e.g., ‘I am very sensitive for pain’) rated between 0 (‘never’) and 5 (‘always’).

#### 2. Fatigue questionnaires

*Modified Fatigue Impact Scale (FIS)*. The MFIS is a self-report instrument designed to rate the extent to which fatigue affects perceived function over the preceding one-week time interval. It includes 3 subscales: cognitive functioning (10 items), physical functioning (10 items), and psychosocial functioning (20 items). Each item is rated on a scale from 0 (‘no problem’) to 4 (‘extreme problem’) with a maximum score of 120 [22].

#### 3. Mood questionnaires

*Beck Depression Inventory (BDI-II)*. The BDI is a questionnaire to evaluate the severity of depressive mood states. The BDI scores severity of components such as feelings of hopelessness and guilt in addition to fatigue and other physical symptoms [23]. It consists of 21 questions, rated between 0 (no symptom impact) and 3 (maximum symptom impact) with a maximum score of 63.

### Electrophysiological data

#### 1. EEG acquisition and preprocessing

EEG recordings were obtained in a fully lighted room. Each participant was sitting upright on a small but comfortable chair. The recording lasted approximately five minutes. The EEG was sampled using Mitsar-201 amplifiers (NovaTech http://www.novatecheeg.com/) with 19 electrodes placed according to the standard 10–20 International placement (Fp1, Fp2, F7, F3, Fz, F4, F8, T7, C3, Cz, C4, T8, P7, P3, Pz, P4, P8, O1, O2), analogous to what was done in the control group. We checked the impedances to remain below 5 kΩ. Data was collected with eyes-closed with a sampling rate of 500 Hz and band passed in the range of 0.15-200Hz. The data was band-pass filtered off-line in the range 2–44 Hz, resampled to 128 Hz and subsequently transposed into Eureka! software [24]. The data were plotted, and carefully inspected for manual artifact-rejection. We removed for the stream of the EEG. All episodic artifacts including eye blinks, eye movements, teeth clenching, body movement, or ECG artifact were removed. Average Fourier cross-spectral matrices were computed for frequency bands delta (2–3.5 Hz), theta (4–7.5 Hz), alpha1 (8–10 Hz), alpha2 (10-12Hz), beta1 (13–18 Hz), beta2 (18.5–21 Hz), beta3 (21.5–30 Hz), and gamma (30.5–44 Hz).

#### 2. Source localization

To estimate the standardized low-resolution brain electromagnetic tomography (sLORETA; Pascual-Marqui, 2002) was used the intracerebral electrical sources that generated the recorded activity (at sensor level). The common average reference transformation [25] was performed before applying the sLORETA algorithm and computing the electric neuronal activity as current density (A/m2). The solution space used in this study and associated leadfield matrix are those implemented in the LORETA-Key software (freely available at http://www.uzh.ch/keyinst/loreta.htm). The software implements the realistic electrode coordinates [26] and the lead field produced by Fuchs et al. (2002) applying the boundary element method on the MNI-152 (Montreal neurological institute, Canada) template of Mazziotta et al. [27]. The sLORETA-key anatomical template divides and labels the neocortical (including hippocampus and anterior cingulated cortex) MNI-152 volume in 6,239 voxels of dimension 5 mm^3^, based on probabilities returned by the Demon Atlas [28]. The co-registration makes use of the correct translation from the MNI-152 space into the Talairach and Tournoux space [29].

#### 3. Lagged phase connectivity

Coherence and phase synchronization between time series corresponding to different spatial locations are interpreted as indicators of the “functional connectivity”. Due to this is highly contaminated with an instantaneous, non-physiological contribution due to volume conductionPascual-Marqui [30] introduced a new measure of coherence by taking into account only non-instantaneous (lagged) connectivity. Hence, effectively removing the confounding factor of volume conduction. As such, this measure of dependence can be applied to any number of brain areas jointly (i.e., distributed cortical networks, whose activity can be estimated with sLORETA). Measures of linear dependence (coherence) between the multivariate time series are defined. The measures are non-negative, taking the value zero only when there is independence, and are defined in the frequency domain: delta (2–3.5 Hz), theta (4–7.5 Hz), alpha1 (8–10 Hz), alpha2 (10-12Hz), beta1 (13–18 Hz), beta2 (18.5–21 Hz), beta3 (21.5–30 Hz), and gamma (30.5–44 Hz). Based on this principle, lagged linear connectivity was calculated. Time-series of current density were extracted for different region of interests using sLORETA. Power in all 6,239 voxels was normalized to a power of 1 and log-transformed at each time point. Region of interest values thus reflect the log-transformed fraction of total power across all voxels and do so separately for specific frequencies. Regions of interest were defined based upon all brain areas obtained in previous analyses for the different frequencies. We include the left and right insula (BA13), the dorsal anterior cingulate cortex (BA24), the left and right dorsal lateral prefrontal cortices (BA9), and the posterior cingulate cortex (BA23).

### Statistical analysis

Statistical analysis is based on estimating, via randomization, the empirical probability distribution for the max-statistic under the null hypothesis comparisons [31]. This methodology corrects for multiple testing (i.e., for the collection of tests performed for all voxels and for all frequency bands). Due to the non-parametric nature of the method, its validity does not rely on any assumption of Gaussianity [31]. sLORETA statistical contrast maps were calculated through multiple voxel-by-voxel comparisons in a logarithm of F-ratio [30]. The significance threshold is based on a permutation test with 5000 permutations [30].

In addition, a similar technique was applied for connectivity. Connectivity contrast maps were calculated through multiple comparisons using t-statistics. The significance threshold was based on a permutation test with 5000 permutations.

A correlation analysis for the fibromyalgia patients, was conducted between the logged transformed multiple voxel-by-voxel comparisons current density of sources for the 8 frequency bands and respectively the FIQ, PVAQ, BDI, and FIS. A permutation testing with 5000 permutations was used to define the significance threshold and to correct for multiple comparisons.

A similar analysis was applied for the connectivity strength between each region of interest for the 8 frequency bands and respectively the FIQ, PVAQ, BDI, and FIS. A permutation testing with 5000 permutations was used to define the significance threshold and to correct for multiple comparisons.

## Results

### Behavioral data for fibromyalgia patients

The average of duration of the illness was 14.87 months (*Sd* = 11.45). The Fibromyalgia impact questionnaire (FIQ) total score was 71.06 (*Sd* = 26.25) and the pain vigilance and awareness questionnaire (PVAQ) score was 40.59 (*Sd* = 12.41). The score for the FIS was 59.72 (*Sd* = 14.57). The Beck depression inventory score was 23.90 (*Sd* = 11.03).

### Brain activity

#### 1. Fibromyalgia vs healthy controls subjects

**Fig 1:**
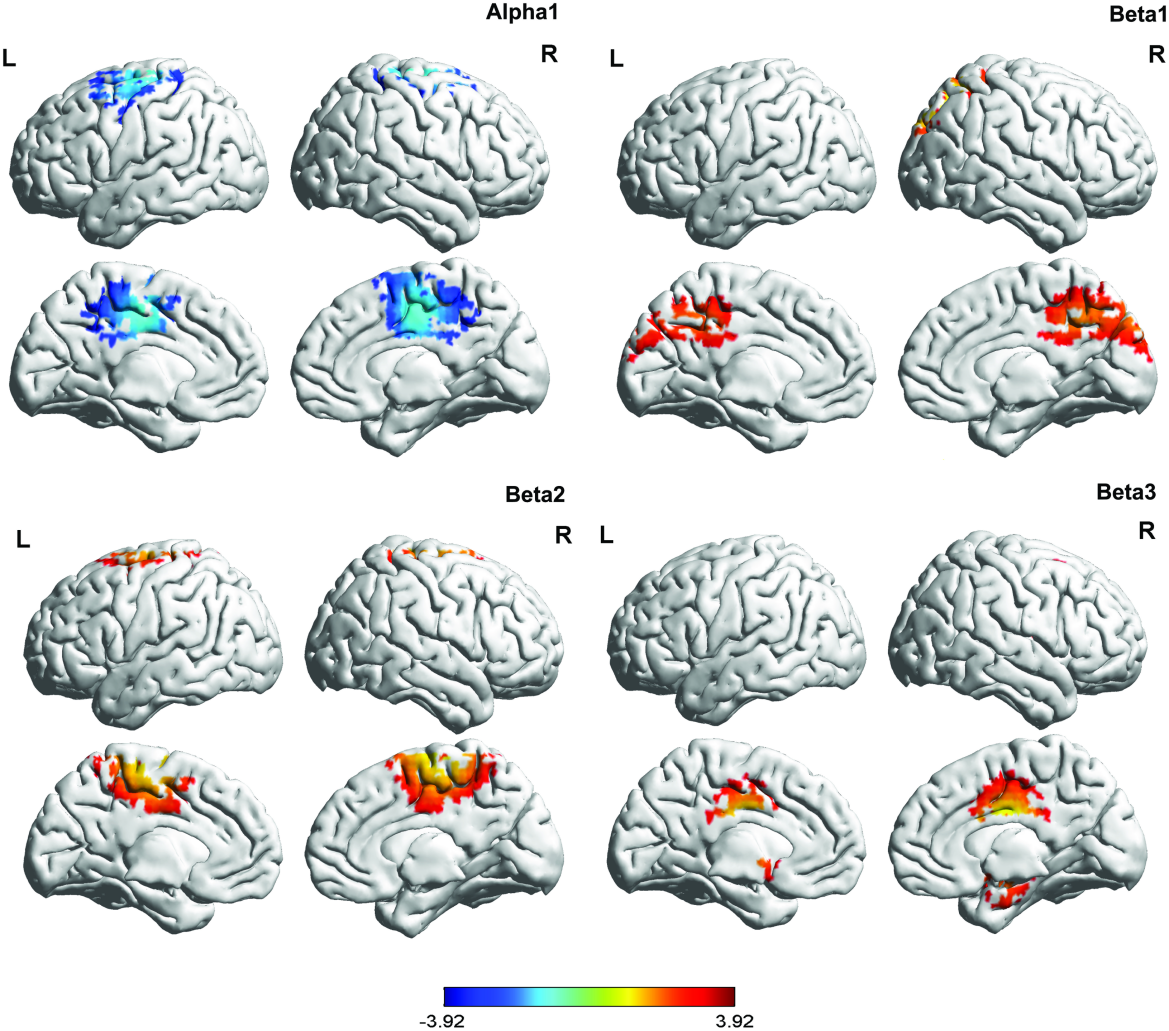
A comparison between fibromyalgia patients and healthy controls. For the alpha1 frequency, a decrease in activity was identified in the posterior cingulate cortex extending into to the precuneus for the fibromyalgia patients. For the beta1 and beta2 frequency bands, increased spontaneous activity was measured within the posterior cingulate cortex extending into the precuneus. In addition, a significant effect was found for the beta3 frequency band revealing increased activity in the dorsal anterior cingulate cortex and the subgenual anterior cingulate cortex.

A comparison between fibromyalgia patients and healthy controls revealed a significant effect for the alpha1, beta1, beta2, and beta3 frequency bands (see Table 1 & Fig 1). For the alpha1 frequency, a decrease in activity in the posterior cingulate cortex extending into the precuneus was found for the fibromyalgia patients. For the beta1 and beta2 frequency bands, increased spontaneous activity was measured within the posterior cingulate cortex extending into the precuneus. In addition, increased beta3 activity in the dorsal anterior cingulate cortex and the subgenual anterior cingulate cortex was found. No significant effects were obtained for the delta, theta, alpha2, or gamma frequency bands.

**Table 1:** MNI coordinates and Brodmann of peak voxels for each cluster.

Analysis	Frequency Band	MNI coordinate			Brodmann area	Name	t-value
		x	y	z			
*Fibromyalgia patients versus Healthy control subjects*	Alpha1	-15	-15	-50	31	Posterior Cingulate Cortex	3.46
	Beta1	0	-36	37	31	Posterior Cingulate Cortex	3.82
	Beta2	-5	-25	44	31	Posterior Cingulate Cortex	3.78
	Beta3	-65	-24	17	24	Dorsal Anterior Cingulate Cortex	3.90
	Beta3	-1	2	-5		Subgenual Anterior Cingulate Cortex	3.25
Correlation FIQ							*r*
	Alpha1	5	30	25	32	Dorsal Anterior Cingulate Cortex	.34
	Beta1	5	25	40	32	Dorsal Anterior Cingulate Cortex	.34
Correlation PVAQ							*r*
	Alpha1	5	30	25	32	Pregenual Anterior Cingulate Cortex	.34
	Beta1	5	25	40	32	Pregenual Anterior Cingulate Cortex	.34
Correlation BDI	Alpha2	5	25	27	24	Dorsal Anterior Cingulate Cortex	.44
	Beta3	-5	25	15	32	Dorsal Anterior Cingulate Cortex	.41
	Beta3	-5	19	-7	25	Subgenual Anterior Cingulate Cortex	.39
Correlation BDI	Theta	-5	30	35	24	Dorsal Anterior Cingulate Cortex	.34
	Gamma	-10	20	25	32	Dorsal Anterior Cingulate Cortex	.41
	Gamma	-10	12	-15	25	Subgenual Anterior Cingulate Cortex	.35

#### 2. Correlation between brain activity and the FIQ in patients with fibromyalgia

**Fig 2:**
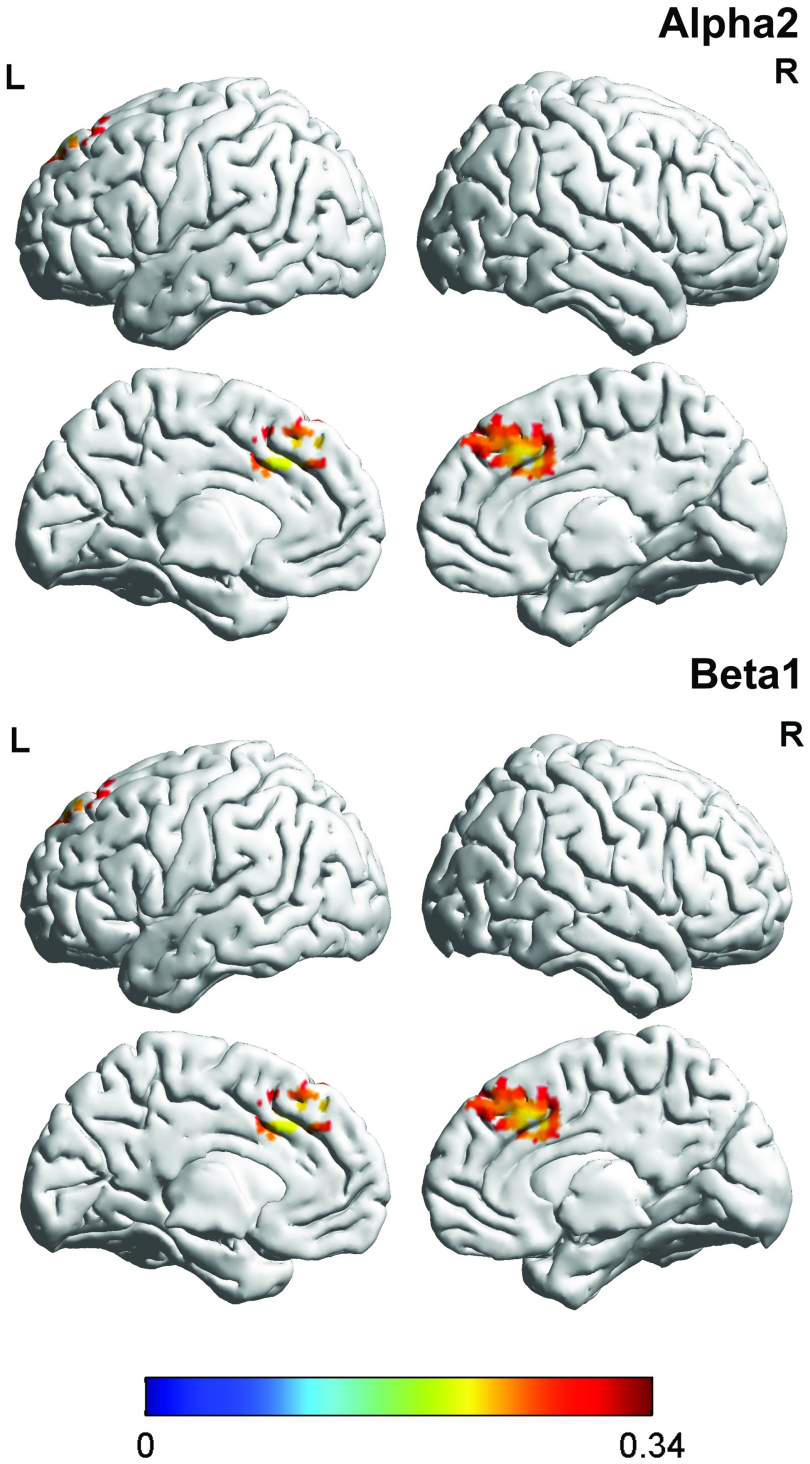
Correlation with FIQ. For the alpha2 and beta1 frequency bands, a positive correlation was identified within the dorsal anterior cingulate cortex: the higher the current density in the dorsal anterior cingulate cortex for the alpha1 and beta1 frequency, the higher the score on the FIQ and vice versa.

For the patients with fibromyalgia, a correlation analysis between the FIQ and brain activity revealed a significant effect for the alpha 2 and beta1 frequency bands (see Table 1 & Fig 2). For both frequency bands, a positive correlation was found in the dorsal anterior cingulate cortex indicating that the higher the current density in the dorsal anterior cingulate cortex for the alpha1 and beta1 frequency are, the higher the score on the FIQ and vice versa. No significant effects were obtained for the delta, theta, alpha1, beta2, beta3, or gamma frequency bands.

#### 3. Correlation between brain activity and the PVAQ in patients with fibromyalgia

**Fig 3:**
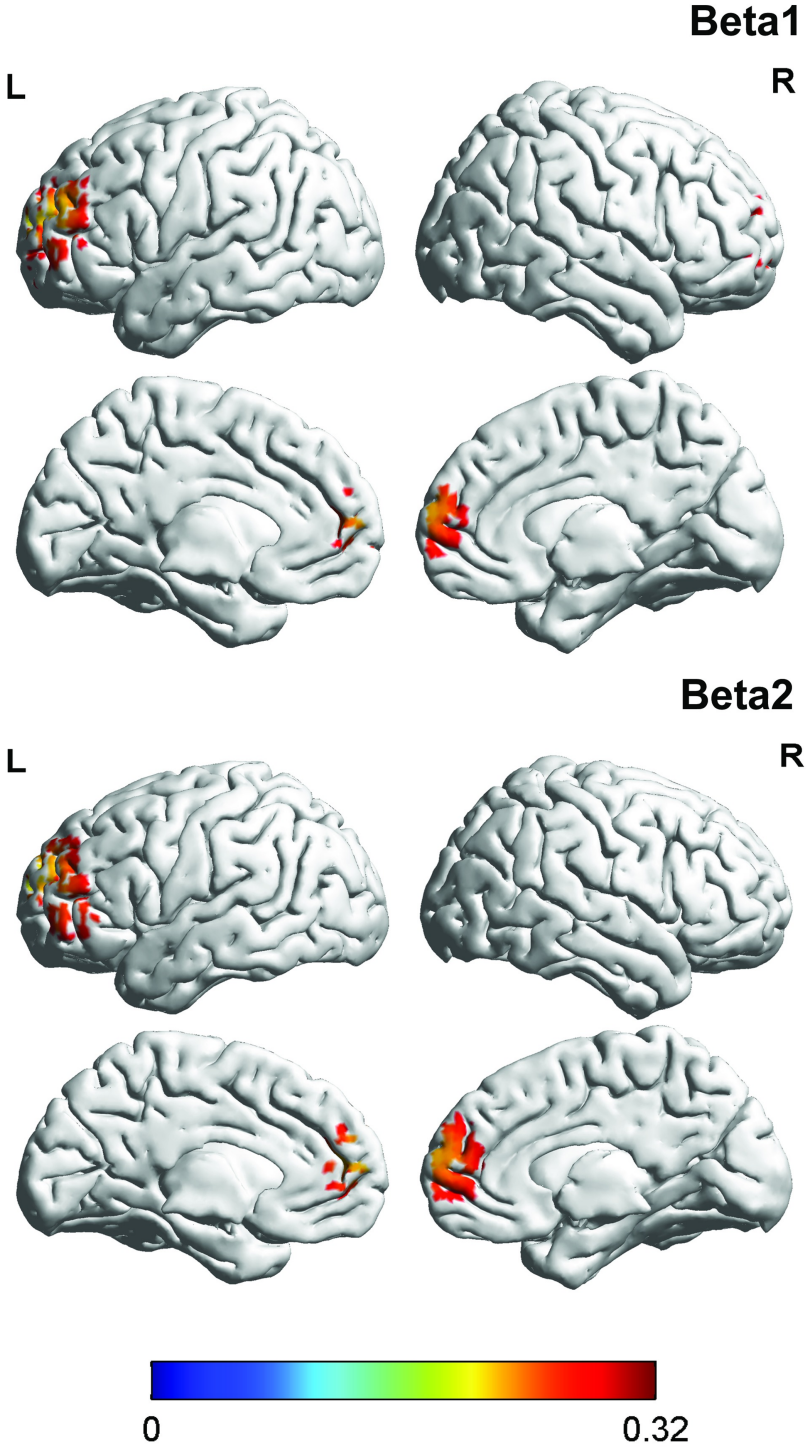
Correlation with PVAQ. A significant effect was demonstrated for the beta1 and beta2 frequency band in both the pregenual anterior cingulate extending into the dorsal medial/lateral prefrontal cortex with the PVAQ and vice versa.

A correlation analysis between the PVAQ and brain activity was also computed. A significant effect was demonstrated for both the beta1 and beta2 frequency bands in the pregenual anterior cingulate extending into the dorsal medial/lateral prefrontal cortex (see Table 1 & Fig 3). These effects revealed that the higher patients scored on the PVAQ, the higher the current density was within the pregenual anterior cingulate extending into the dorsal medial/lateral prefrontal cortex for the beta1 and beta2 frequency bands and vice versa. No significant effects were obtained for the delta, theta, alpha1, alpha2, beta3, or gamma frequency bands.

#### 4. Correlation between brain activity and the BDI in patients with fibromyalgia

**Fig 4:**
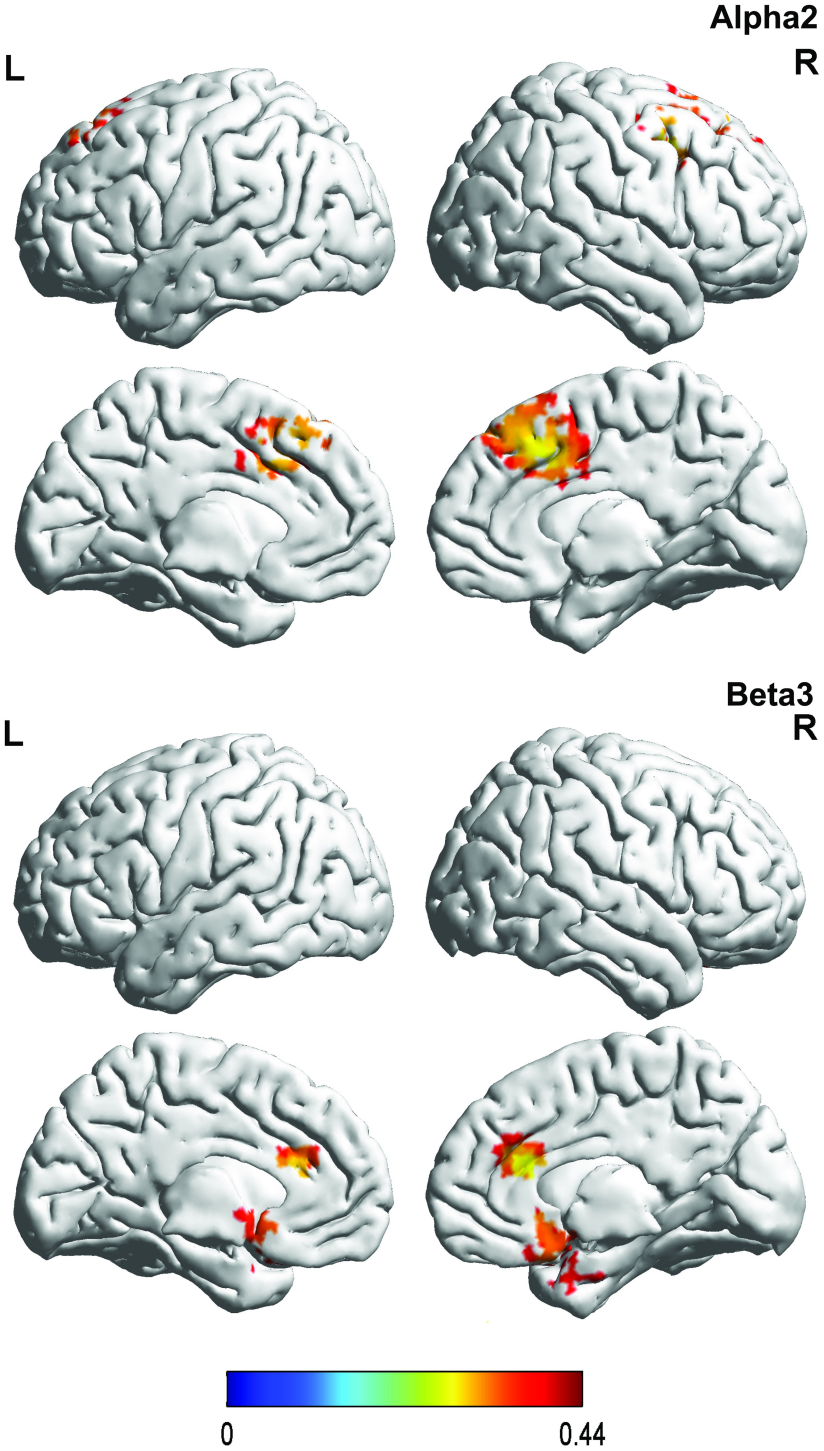
Correlation with BDI. For the alpha2 frequency band, a positive correlation was identified in the dorsal anterior cingulate cortex, while for the beta 3 frequency band a positive correlation was observed in both dorsal anterior cingulate cortex and the subgenual anterior cingulate cortex.

We performed a correlation analysis between the BDI and brain activity which demonstrated a significant correlation for the alpha2 band in the dorsal anterior cingulate cortex and for the beta3 band in the dorsal ACC and pgACC (see Table 1 & Fig 4). No significant effects were obtained for the delta, theta, alpha1, beta1, beta2, or gamma frequency bands.

#### 5. Correlation between brain activity and the FIS in patients with fibromyalgia

**Fig 5:**
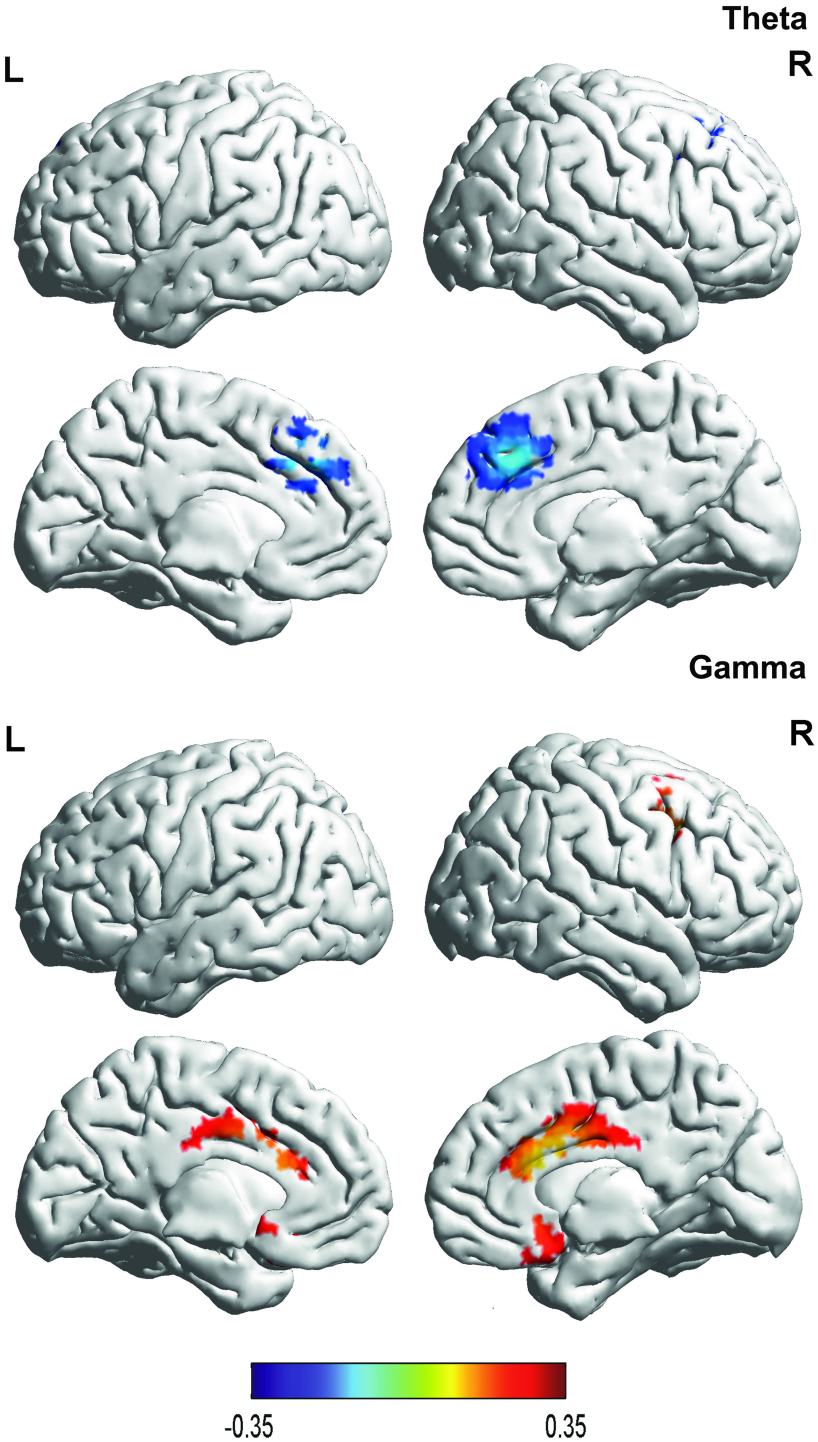
Correlation with FIS. For the theta frequency band, a negative correlation was found between the current density in the dorsal anterior cingulate cortex and the FIS, while for the gamma frequency band positive correlations were demonstrated between the subgenual anterior cingulate cortex, dorsal anterior cingulate cortex, and the FIS respectively.

A correlation analysis revealed a significant correlation for the theta and gamma frequency bands based on the FIS with brain activity (see Table 1 & Fig 5). For the theta frequency band, a negative correlation was obtained between the current density in the dorsal anterior cingulate cortex and the FIS, while for the gamma frequency band, positive correlations were demonstrated between the subgenual anterior cingulate cortex, dorsal anterior cingulate cortex, and the FIS respectively. No significant effects were obtained for the delta, alpha1, alpha2, beta1, beta2, or beta3 frequency bands.

### Brain connectivity

#### 1. Fibromyalgia vs healthy controls subjects

**Fig 6:**
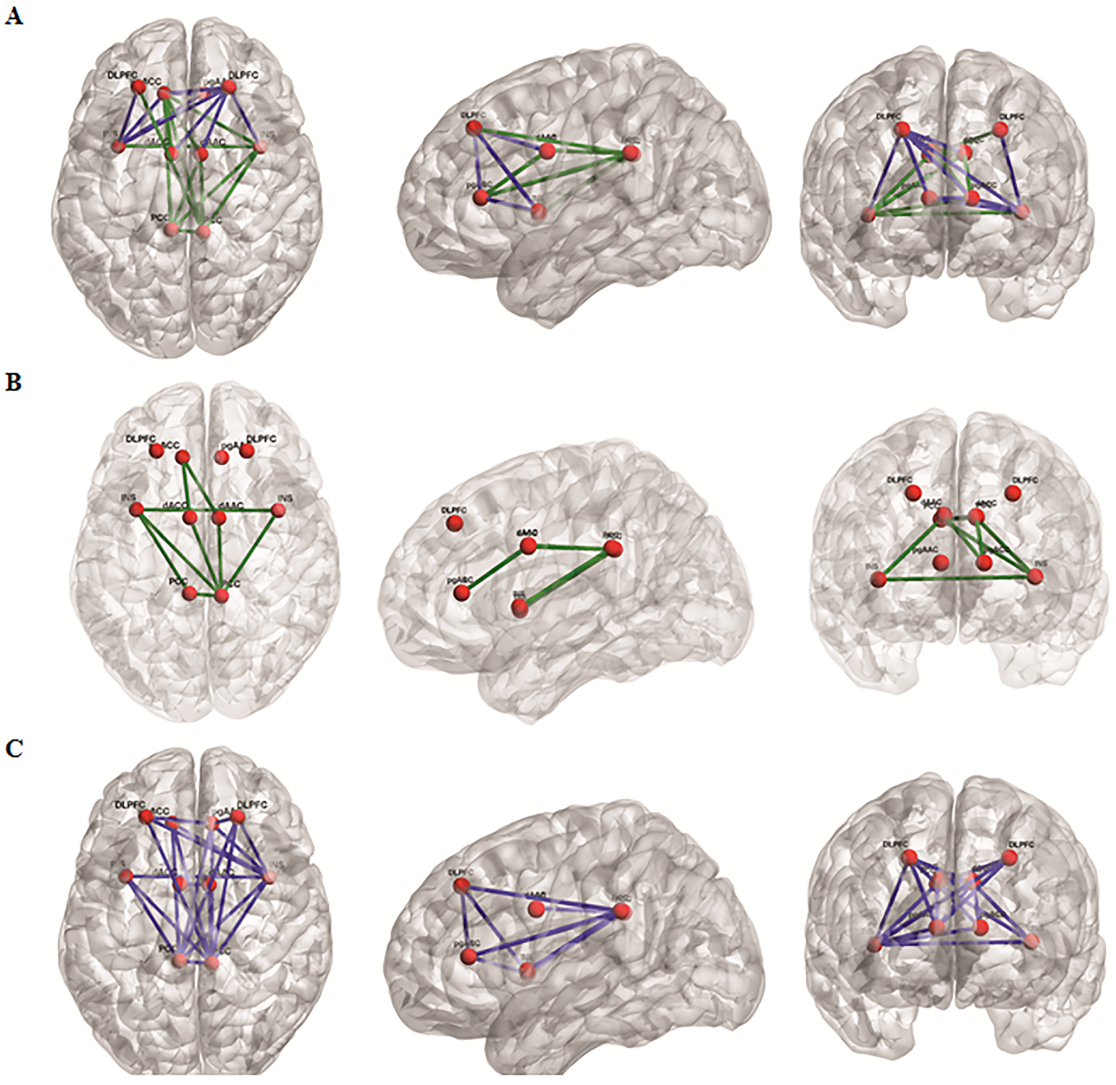
(A) A comparison between fibromyalgia patients and healthy controls. For the alpha2 frequency band, decreased connectivity (blue lines) was identified between the right dorsal lateral prefrontal cortex and multiple areas, including the right and left insula, the right and left pregenual anterior cingulate cortex, and the right and left dorsal anterior cingulate cortex. There was also a decrease in functional connectivity in the alpha2 frequency band between the left insula and the left and right dorsal lateral prefrontal cortex and the left and pregenual anterior cingulate. Increased functional connectivity (green lines) was found between the right insula and multiple areas, including the left insula, left pregenual anterior cingulate cortex, and bilateral posterior cingulate cortex for the alpha2 frequency band. An increase in functional connectivity was also found between the left pregenual anterior cingulate cortex and right insula, left and right pregenual anterior cingulate cortex, and left and right posterior cingulate cortex for the alpha2 frequency band. An increase was also noted between the left posterior cingulate cortex and multiple areas, including right posterior cingulate cortex, right insula, right pregenual anterior cingulate cortex, and left pregenual anterior cingulate cortex. A similar increase in functional connectivity for the alpha2 frequency band was also identified between the right posterior cingulate cortex and multiple areas including the right insula and right dorsal anterior cingulate cortex, left PCC, left pregenual anterior cingulate cortex, and left dorsal lateral prefrontal cortex. (B) A correlation between fibromyalgia and FIQ. A positive correlation was found between the FIQ and alpha2 functional connectivity between multiple areas including the left insula, the right insula, and the left and right posterior cingulate cortex, as well as between the left pregenual anterior cingulate cortex and left and right dorsal anterior cingulate cortex, and between the right posterior cingulate cortex and left posterior cingulate cortex, left and right insula, left and right dorsal anterior cingulate cortex. (C) A correlation between fibromyalgia and FIS. A negative correlation was identified between the FIS and functional connectivity between most of the regions of interest, i.e. the dorsal lateral prefrontal cortex, the insula, the pregenual anterior cingulate cortex, the dorsal anterior cingulate cortex, and the posterior cingulate cortex for the delta frequency band.

A comparison of fibromyalgia subjects versus healthy control subjects for functional brain connectivity, as calculated by lagged phase synchronization, specific to the alpha2 frequency band revealed multiple differences (*F* = 3.91, *p* = .001; see Table 2 & Fig 6A). Decreased connectivity was identified between the right dorsal lateral prefrontal cortex and multiple areas, including the left and right insula, the left and right pgACC, and the bilateral anterior cingulate cortex. There was also a decrease in functional connectivity between the left insula and the left pgACC. Increased functional connectivity was found between the right insula and multiple areas, including the left insula, left pgACC, and bilateral posterior cingulate cortex. An increase in functional connectivity was also found between the left pgACC and bilateral dorsal anterior cingulate cortex, and bilateral posterior cingulate cortex. An increase was also noted between the left posterior cingulate cortex and multiple areas including the right posterior cingulate cortex, right insula, right dorsal anterior cingulate cortex, and left pregenual anterior cingulate. A similar increase in functional connectivity was also found between the right posterior cingulate cortex and multiple areas including the right insula, right dorsal anterior cingulate cortex, left pgACC, and left dorsal lateral prefrontal cortex. No significant effects were obtained for the delta, theta, alpha1, beta1, beta2, beta3, or gamma frequency bands.

**Table 2:** Connectivity analysis.

Analysis	Frequency Band	Connectivity	Name	*F*-Value
*Fibromyalgia patients versus Healthy control subjects*	Alpha2	Increase	right INS ↔ left INS; right INS ↔ left pgACC; right INS ↔ bil PCC; left pgACC ↔ dACC; pgACC ↔ bil PCC; left PCC ↔ right PCC; left PCC ↔ right INS; left PCC ↔ right dACC; left PCC ↔ left pgACC; right PCC ↔ right INS; left PCC ↔ right dACC; left PCC ↔ left pgACC; left PCC ↔ left DLPFC	3.91
	Alpha2	Decrease	right DLPFC ↔ left INS; Right DLPFC ↔ right INS; right DLPFC ↔ left pgACC, right DLPFC ↔ right pgACC, right DLPFC ↔ dACC; left INS ↔ bil pgACC	3.91
Correlation FIQ				*r*
	Alpha2	Positive	right INS ↔ left INS; right INS ↔ right PCC; right PCC ↔ left PCC; right PCC ↔ right dACC; right PCC ↔left dACC; left PCC ↔ left INS; left pgACC ↔ left dACC; left pgACC ↔ right dACC	.39
Correlation PVAQ	-			
Correlation BDI	-			
Correlation BDI	Delta	Negative	right DLPFC ↔ right INS; right DLPFC ↔ left pCC; right DLPFC ↔ right PCC; right DLPFC ↔ right pgACC; left DLPFC ↔ right pgACC; right DLPFC ↔ right INS; right DLPFC ↔ right PCC; left pgACC ↔ right pgACC; left pgACC ↔ left PCC; left pgACC ↔ right PCC; right pgACC ↔ right INS; right pgACC ↔ left PCC; right pgACC ↔right PCC; right dACC ↔ right PCC; right dACC ↔ left PCC; leftt dACC ↔ right PCC; left dACC ↔ left PCC; right INS ↔ right PCC; right INS ↔ left PCC; left INS ↔ right PCC; left INS ↔ left PCC	-.41

#### 2. Correlation between brain connectivity and the FIQ in patients with fibromyalgia

A positive significant correlation was revealed between connectivity and patient`s individual score on the FIQ for the alpha2 frequency band (r = .39, *p* < .05; see Table 2 & Fig 6B). This positive correlation was found between the patient`s individual score on FIQ and functional connectivity between multiple areas including the left and right insula, the left and right posterior cingulate cortex, the left pgACC, and left and right dorsal anterior cingulate cortex, between the left and right posterior cingulate cortex, and the left and right dorsal anterior cingulate cortex. In other words, the higher the score on the FIQ, the higher the lagged phase connectivity strength between these regions and vice versa. No significant effects were obtained for the delta, theta, alpha1, beta1, beta2, beta3, or gamma frequency bands.

#### 3. Correlation between brain connectivity and the PVAQ in patients with fibromyalgia

A correlation analysis between brain connectivity and patient`s individual score on PVAQ revealed no significant effects for the delta, theta, alpha1, alpha2, beta1, beta2, beta3, or gamma frequency bands.

#### 4. Correlation between brain connectivity and the BDI in patients with fibromyalgia

A correlation analysis between brain connectivity and patient`s individual score on BDI revealed no significant effects for the delta, theta, alpha1, alpha2, beta1, beta2, beta3, or gamma frequency bands.

#### 5. Correlation between brain connectivity and the FIS in patients with fibromyalgia

A negative correlation was identified between the functional connectivity and patient`s individual score on FIS for the delta frequency band (r = -.41, *p* < .05; see Table 2 & Fig 6C). A negative correlation was obtained between patient`s individual score on FIS and functional connectivity between most of the included areas, namely the dorsal lateral prefrontal cortex, the insula, the pgACC, the dorsal anterior cingulate cortex, and the posterior cingulate cortex. Thus, the higher the score on the FIS, the lower the lagged phase connectivity strength between these regions. No significant effects were obtained for the theta, alpha1, alpha2, beta1, beta2, beta3, or gamma frequency bands.

## Discussion

We have investigated the association between different clinical symptoms of fibromyalgia, including pain, fatigue, mood measures, resting state source localized EEG brain activity, and functional connectivity. Multiple changes were identified in brain activity that correlate with pain, fatigue, and mood questionnaires. Based on the connectivity analysis, our results suggest a strong association with increased connectivity between the posterior cingulate cortex, dorsal anterior cingulate cortex, dorsolateral prefrontal cortex, and pgACC. Also, a decreased connectivity between the dorsal lateral prefrontal network and the dorsal anterior cingulate cortex as well as the pgACC is noted. In addition, the connectivity between the dorsal anterior cingulate cortex and posterior cingulate cortex as well as the dorsal anterior cingulate cortex and the pgACC correlate positively with the fibromyalgia questionnaire, while the connectivity between the dorsal anterior cingulate cortex and posterior cingulate cortex correlates negatively with the fatigue questionnaire.

The pgACC is part of the descending inhibitory pathway and both structural and functional changes, as well as changes in connectivity have been identified in fibromyalgia patients [10, 17, 32]. In this study, a positive correlation between the pgACC and the pain vigilance and awareness questionnaire was found, indicating that the higher the score for pain vigilance and awareness, the higher the activity in the pgACC in the beta frequency range. Since the pgACC is a key region involved in the descending inhibition of pain, activity and connectivity changes in the pgACC in the fibromyalgia patients provides support for the hypothesis that the development of fibromyalgia in patients may be associated with dysfunction of descending pain modulation. There is a distinct overlap between decreased cortical thickness, decreased brain volumes, and decreased functional regional coherence in the pgACC in fibromyalgia [4]. The same area is also overactive resting state in alpha. Persistent beta activity in a pathological state is proposed to result in an abnormal persistence of the status quo and a deterioration of flexible behavioral and cognitive control [33]. This could signify the dysfunctional pain inhibition is maintained and non-adaptive.

It is known that the unpleasantness of pain correlates with activity in the dorsal anterior cingulate cortex [34] and motor responses to pain correlate with subgenual anterior cingulate cortex activity [35]. The dorsal and subgenual anterior cingulate cortex are related to distress in (social) pain [36], analogous to what has been found in tinnitus [37], which is the auditory analogue of pain [38]. Our findings are supported by the idea that these areas are involved in the affective component of pain, as both areas correlate positively with the depression inventory for the alpha and beta range. It is further known that cingulotomy decreases the affective response to noxious stimuli, but does not alter the discriminatory components of pain such as the perceived intensity of the pain [39] or the ability to localize the unpleasant stimulus [40]. In addition, the dorsal and subgenual anterior cingulate cortices were correlated to the fatigue scale for the theta and gamma frequency bands. The persistent co-localized activity of theta and gamma band has been called thalamocortical dysrhythmia and has been shown to be present in the subgenual anterior cingulate cortex [41]. Furthermore, it is known that fatigue and depression are correlated, which was again confirmed in this study (r = .59). Our results also indicate that for the alpha and beta frequency bands, dorsal anterior cingulate activity correlates positively with fibromyalgia impact. Previous research on pain has reported that the alpha and beta rhythms are correlated to both transient and tonic noxious painful experiences [42–45] and might reflect individuals’ inherent tonic pain responsiveness [46]. In addition, neuropathic pain models in animals have demonstrated structural micro- and macro-changes in the anterior cingulate cortex associated with the induction of anxiety-like behavior and attention deficits [47]. In association with the insula, the dorsal anterior cingulate cortex is known to be involved in evaluative processing and forms part of the resting-state salience network [8] which is proposed to evaluate the behavioral relevance of internal states and of incoming external stimuli. As mentioned, the dorsal anterior cingulate cortex and insula are part of the pain matrix, which has been shown to be a multimodal salience network [7]. Increased functional coupling within the salience network has been implicated in emotional salience monitoring and precedes the perception of stimuli as painful [48].

The posterior cingulate gyrus is the central hub of the default mode network, which is anticorrelated to the salience network [49]. Therefore, when no salient external stimuli, such as pain stimuli, are perceived, the default mode is activated. When behaviorally important external stimuli are presented, the salience network is activated and the default mode inactivated [50]. The posterior cingulate cortex is involved in self-referential processing and mind-wandering (day-dreaming, i.e. thinking about the future and reflecting about the past) [51]. Our data are in keeping with previous findings by indicating activity changes in the alpha and the beta frequency bands for fibromyalgia patients in comparison to healthy controls. The posterior cingulate cortex has previously indeed been related to fibromyalgia pain [52]. In addition, changes in the posterior cingulate cortex have been associated with impaired cognition in fibromyalgia [11, 12, 53]. In this study, we found an increase in functional connectivity between the salience network and the default mode network. Previous research already identified a correlation between pain severity and abnormal functional connectivity between the salience network and the default mode network [15], and reduced connectivity between the salience network and the default mode network is correlated with a reduction in pain for fibromyalgia patients [14]. These findings are similar to what was found in chronic back pain [16] as well as in previous fibromyalgia studies [54].

Most of the connectivity effects obtained were related to the alpha2 frequency band. This fits with recent research demonstrating that alpha phase-synchronization or phase-coherence, are a mechanism for short and long-range communication in the brain [55, 56]. The alpha band synchronization reflect a functional mechanism of attention and consciousness that have been linked between default mode structures [57–59]. Furthermore, it was shown that the upper alpha band (10–12 Hz) oscillations has been associated with tonic alertness in a network comprising dorsal anterior cingulate cortex, anterior insula and the anterior prefrontal cortex [59]. The functional connectivity between the posterior cingulate cortex and dorsal anterior cingulate cortex in the alpha frequency band could suggest persistent salience to an unchanging pain stimulus, causing the pain to become part of the self-referential network. In other words, the painful state becomes the norm, a mechanism also known as allostatic reference resetting [60], which is a mechanism that has been hypothesized to occur in fibromyalgia [61]. Allostasis is defined as stability through change and is more efficient than homeostasis by anticipating needs and preparing to satisfy them before they arise [60]. The advantages are obvious and related to a central organ that is capable of prediction—the brain—and therefore can organize concerted mechanisms to maintain adaptive stability [60]. In order to predict, a self-reference or self-perception is essential to interpret the incoming internal and external stimuli [62]. This fits with the idea that in fibromyalgia, pain prediction is dysfunctional but pain processing per se is not [63]. The status quo beta activity found in the pgACC in this study might be an electrophysiological marker for this dysfunctional mechanism.

The anti-correlated activity between an externally oriented network and the default mode network, which is internally oriented [49], has been further refined. The dorsal lateral prefrontal cortex and the salience network exert an inhibitory effect on the default mode network associated with anti-correlated activity. Additionally, the salience network acts as a switch between the central executive network and the default mode networks [64]. The inhibitory effect is mediated via a functional connection between the dorsal lateral prefrontal cortex and the pgACC as demonstrated by transcranial magnetic stimulation and transcranial direct current stimulation [65]. In this fibromyalgia study, we did find that the functional connectivity between the right dorsal lateral prefrontal cortex and bilateral pgACC is decreased.

A limitation of this study is the low resolution of the source localization inherently resulting from a limited number of sensors (19 electrodes). This is sufficient for source reconstruction but results in greater uncertainty in source localization and decreased anatomical precision, and thus the spatial precision of the present study is considerably lower than that of functional MRI. Nevertheless, the tomography sLORETA provides has received considerable validation from studies combining LORETA with other more established localization methods, such as functional Magnetic Resonance Imaging (fMRI) [66, 67], structural MRI [68], and Positron Emission Tomography (PET) [69–71] and was used in previous studies to detect activity in deeper structures, for example [72–74]. Further sLORETA validation has been based on accepting as ground truth the localization findings obtained from invasive, implanted depth electrodes, in which case there are several studies on epilepsy [75, 76] and cognitive ERPs [77]. It is worth emphasizing that deep structures such as the anterior cingulate cortex [78] and mesial temporal lobes [79] can be correctly localized with these methods. The involvement of the deeper structures was already illustrated in previous research using low density EEG and was confirmed subsequently by PET and MRI suggesting the reliabilities of our findings. However, further research could improve spatial precision and accuracy could be achieved using high-density EEG (e.g., 128 or 256 electrodes), subject-specific head models, and MEG recordings. In addition, lagged phase coherence analysis requires the selection of regions of interest based on a priori knowledge, or by means of heuristic procedures (i.e. only analyze functional connectivity between areas with altered activity)[80, 81]. The impossibility of a purely data driven approach (i.e. whole brain connectivity analysis independent of regions of interest) automatically implies a theoretically funded approach is required, thereby selecting regions of interest. This can be seen as a weakness but is unfortunately inherent to the technique [82].

## Conclusion

Our data highlights the functional dynamics of brain regions integrated in brain networks in fibromyalgia patients. Overall, this study supports an important role of the pgACC [4] and also suggests that the degree of activation and the degree of integration within different networks is important. The inhibition of the dorsal lateral prefrontal cortex with the hubs of the default mode network and pain inhibitory pathway seems to be limited by a decreased functional connectivity with the pgACC. This decreased impact of the pgACC leads to spontaneous pain, which—combined with increased functional connectivity between the salience network and the default mode network—may lead to the pain becoming an integrated part of the self-referential default mode network via allostatic reference resetting, causing the painful state to become the norm. The findings of this study suggest that intrinsic brain connectivity is a candidate as an objective marker that is sensitive enough to track pain levels in fibromyalgia. Intrinsic connectivity could therefore potentially be used as a complementary objective outcome measure in longitudinal clinical trials to objectively measure effects of different therapies in this fibromyalgia population.
